# Laparoscopic Sleeve Gastrectomy versus Laparoscopic Banded Sleeve Gastrectomy: First Prospective Pilot Randomized Study

**DOI:** 10.1155/2016/6419603

**Published:** 2016-04-10

**Authors:** Valeria Tognoni, Domenico Benavoli, Emanuela Bianciardi, Federico Perrone, Simona Ippoliti, Achille Gaspari, Paolo Gentileschi

**Affiliations:** ^1^Bariatric Surgery Unit, Department of Experimental Medicine and Surgery, University of Rome “Tor Vergata”, Viale Oxford 81, 00133 Rome, Italy; ^2^Psychiatric Unit, Department of System Medicine, University of Rome “Tor Vergata”, Viale Oxford 81, 00133 Rome, Italy

## Abstract

*Introduction*. The placement of ring or band around the gastric tube might prevent the dilation after Laparoscopic Sleeve Gastrectomy (LSG). We describe the first randomized study comparing LSG and Laparoscopic Banded Sleeve Gastrectomy (LBSG).* Material and Method*. Fifty obese patients were enrolled in the study between January 2014 and January 2015. We analysed differences in operative time, complication rate, mortality, and BMI between the two groups over a period of 12 months.* Results*. Twenty-five patients received LSG (group A) and 25 LBSG (group B). The mean preoperative BMI was 47.3 ± 6.58 kg/m^2^ and 44.95 ± 5.85 kg/m^2^, respectively, in the two groups. There was no statistical relevant difference in operative time. No intraoperative complications occurred. Mean BMI registered after 3, 6, and 12 months in groups A and B, respectively, were 37.86 ± 5.72 kg/m^2^ and 37.58 ± 6.21 kg/m^2^ (*p* = 0.869), 33.64 ± 6.08 kg/m^2^ and 32.03 ± 5.24 kg/m^2^ (*p* = 0.325), and 29.72 ± 4.40 kg/m^2^ and 27.42 ± 4.47 kg/m^2^ (*p* = 0.186); no statistical relevant difference was registered between the two groups.* Conclusion*. LBSG is a safe and feasible procedure. The time required for the device positioning did not influence significantly the surgical time. The results of bodyweight loss did not document any statistically significant differences among the two groups, even though LBSG group showed a mean BMI slightly lower than that of the control group.

## 1. Introduction/Purpose

Laparoscopic Sleeve Gastrectomy (LSG) is one of the most performed bariatric procedures worldwide, second only to Roux-en-Y gastric bypass (RYGB) [1]. Although both surgeries are effective producing weight loss and obesity-related comorbidities resolution, long-term weight regain remains a main issue.

The success of sleeve gastrectomy may be limited by dilation of the remaining gastric tube, thus diminishing the restrictive effect of this operation [2]. This phenomenon has been shown to occur more often in super obese patient with preoperative BMI > 50 kg/m^2^ [2] and after three to five years from the primary surgery.

It usually leads to reoperation as conversion to RYGB or biliopancreatic diversion (BPD) and in selected cases to a resleeve [3].

In an effort to prevent gastric dilation and increase gastric restriction to promote weight loss in the long term, some authors proposed the use of an additional restriction obtained by placing of ring or band around the gastric tube [4–6]. The literature in this regard is scarce with no prospective or randomized studies.

The innovative concept of banding the neogastric tube derived from the promising results achieved previously with banded gastric bypass (BGBP) was proposed as both a redo and primary surgery to treat or to prevent the weight regain after RYGB [7–9].

The bands or rings used in these operations have been usually fashioned by surgeons from various materials as linea alba, fascia lata, Gore-Tex®, Marlex mesh, Silastic tubing, porcine graft, bovine graft, and so forth. Fobi was one of the first authors describing the surgical technique of banded RYGB [10] and subsequently the use of GaBP Ring System*™* as a standard nonabsorbable premanufactured device in order to provide a better standardization and quality control than surgeon-fashioned bands or ring [7].

We describe the preliminary results of the first prospective randomized study comparing two groups of morbidly obese patients undergoing LSG and Laparoscopic Banded Sleeve Gastrectomy (LBSG) using the GaBP Ring Autolock System. This is a pilot randomized trial on a sample of 50 patients and a 1-year follow-up while the long-term (5-years) results and a larger sample of patients will be further evaluated.

The aim of this study was to evaluate the safety and effectiveness of the LBSG in terms of incidence of complications and obesity comorbidity resolution; moreover we aimed to evaluate possible differences in weight loss in terms of BMI reduction between the two groups in the short and long term.

## 2. Material and Method

**Figure 1:**
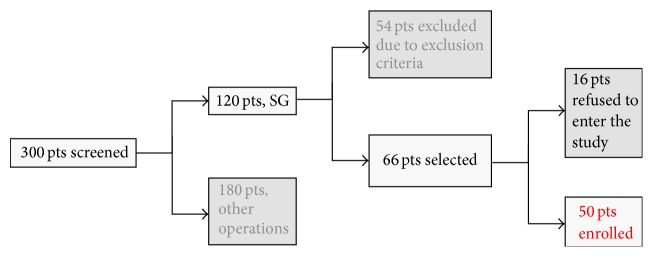
Diagram of patients selection.

This is a prospective randomized trial, carried on in a single bariatric centre. We report the preliminary results obtained with 50 patients at 1-year follow-up. The selection of patients to be included in the study was performed in 2013 (Figure 1). We screened a total of 300 bariatric patients in 2013; of these, 120 patients were selected for SG. Fifty-four patients were excluded from the study due to exclusion criteria as mentioned here below. Of the remaining 66 patients, 50 accepted to enter the study and have been operated on between January 2014 and January 2015.

Exclusion criteria included age <18 or >60 years, previous bariatric or gastrointestinal surgery, psychiatric illness, pregnancy, and absolute contraindications to pneumoperitoneum.

All patients were invited to participate in this study and informed in detail about the risks and the benefits of each operation, and a written informed consent was obtained from all individual participants included in the study.

All procedures performed in studies involving human participants were in accordance with the ethical standards of the institutional and/or national research committee and with the 1964 Helsinki declaration and its later amendments or comparable ethical standards. The study was approved by the local ethics committee.

The randomization was obtained by drawing two opaque envelopes containing, respectively, a card with the indication to LSG or LBSG.

**Figure 2:**
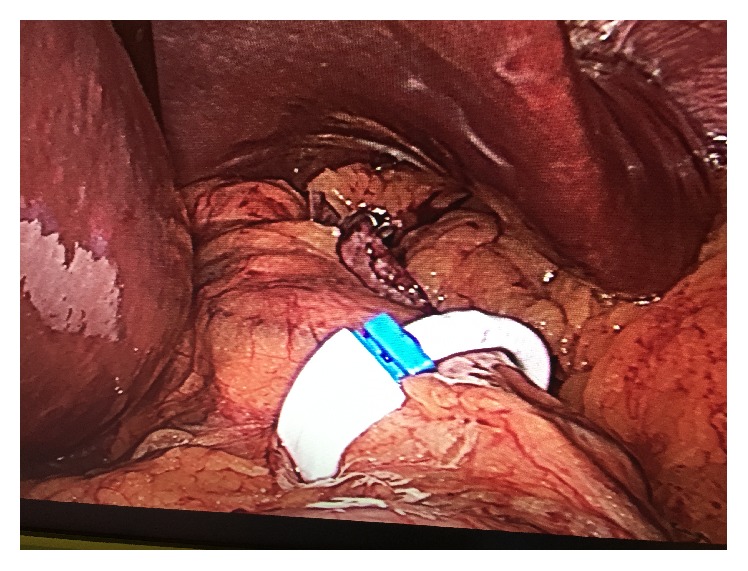
Laparoscopic Sleeve Gastrectomy with GaBP Ring Autolock*™* System.

**Figure 3:**
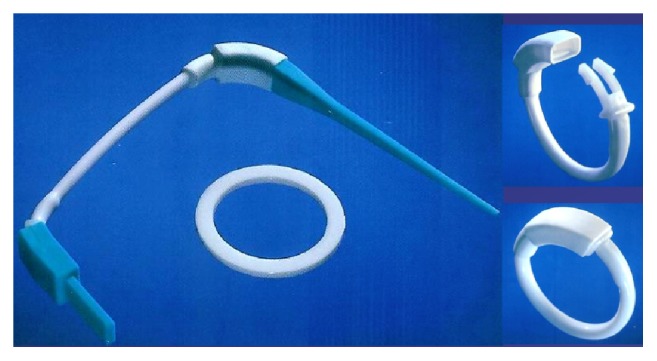
GaBP Ring Autolock System.

An interdisciplinary team evaluated candidates based on a medical, nutritional, endocrinological, and psychiatric work-up. Standard preoperative assessments included barium X-ray of the upper gastrointestinal tract or esophagogastroduodenoscopy, blood examinations, cardiologic evaluation, and chest radiography. Psychiatric counselling was conducted to evaluate mental health contraindications to surgery. All procedures were performed laparoscopically, using four or five ports, by the same surgeon. LSG was performed with 36-F bougie and gastric resection was carried out with a reinforced linear stapler. The ring used was the GaBP Ring Autolock System (Figure 3), composed of a radiopaque silicon coated implantable device with a plastic one-way lock mechanism at the ends of the ring. It was placed 4 cm distal from the cardia hiatus through a retrogastric tunnel created in the pars flaccida of the hepatogastric ligament (Figure 2). The diameter of the ring selected to be used in our study was 7 cm; only two patients received a 7.5 cm ring due to excessive narrowing of the gastric tube.

Every patient underwent an upper gastrointestinal swallow with gastrografin on the second day after surgery.

Preoperative data as BMI, obesity-related comorbidities as hypertension, T2 diabetes mellitus (T2DM), OSAS, and pharmacologic therapy were included in our prospective database. Operative time, adverse event or complication, and hospital stay were also included in the database.

Postoperative advice included a diet consisting of clear liquids and puréed foods for 15 days and a semisolid-consistency diet for the next 15 days. After the first 30 days, patients gradually began a low-fat, low-carbohydrate, high-protein solid diet based on the advice of a dietitian.

The follow-up included evaluation at 15 days and 1, 3, 6, and 12 months after surgery.

Solid food intolerance and remission of hypertension as well as T2DM or OSAS have been evaluated during the follow-up. Hypertension and T2DM resolution have been considered for pressure values <140/80 mmHg and glucose blood value <126 gr/dL after drug therapy suspension, respectively.

This is a preliminary report of a pilot randomized trial focusing on the use of a ring after a SG. Our preliminary primary endpoint was to analyse feasibility and safety of BSG. Primary endpoint of the longer study will be to analyse differences in BMI at 1, 3, 6, and 12 months between the two groups. Secondary endpoint was to evaluate differences in operative time, hospital stay, and postoperative short- and long-term complication.

Statistical analyses were performed using IBM SPSS version 20 for Windows. Categorical variables were analysed using the chi-squared test, Fisher's exact test, or Student's *t*-test for quantitative and qualitative variables, as appropriate. Data are expressed as median and range, unless otherwise specified. *p* values are two sided, and values <0.05 were considered statistically significant. Continuous variables are described as mean and standard deviation (SD), whereas categorical variables were described as number and percentage.

## 3. Results

Fifty obese patients have been enrolled in the study, randomization-assigned 25 pts to group A and group B, respectively. Twenty-five patients (16 women, 9 men) received LSG (group A), and 25 (16 women, 9 men) underwent LBSG (group B).

In group A, the mean age was 43.7 ± 9.8 years and the mean preoperative BMI was 47.3 ± 6.58 kg/m^2^. These values were 45.7 ± 12.7 years and 44.95 ± 5.85 kg/m^2^ in group B.

Twelve patients had preoperatory T2DM, 7 in group A (28%) and 5 in group B (20%), respectively; and 21 patients suffered of hypertension and took antihypertensive drugs, 14 in group A (56%) and 7 in group B (28%). Eight patients had OSAS, 6 in group A and 2 in group B, respectively (see Table 1).

The mean operative time was 74.60 ± 14.48 min and 84.60 ± 30.13 min in the two groups, respectively (*p* = 0.144). No intraoperative complication occurred.

Every patient had a minimum follow-up of 6 months; 28 patients had a follow-up of 12 months, 16 in group A and 12 in group B, respectively.

We registered 3 postoperative complications (Table 2), 2 bleeding incidences, one in each group of patients, managed with blood transfusion and prolonged hospitalization (7 days for the patient receiving LBSG and 5 for the patient undergoing LSG); no reoperation was required.

One patient receiving LSG developed a gastric stenosis. He experienced food intolerance as soon as a solid diet was reintroduced. An esophagogastroduodenoscopy confirmed the presence of the stenosis; the sleeve was converted in RYGB and the patient was excluded from the study.

Mean BMI registered after 3, 6, and 12 months in groups A and B, respectively, was 37.86 ± 5.72 kg/m^2^ and 37.58 ± 6.21 kg/m^2^ (*p* = 0.869), 33.64 ± 6.08 kg/m^2^ and 32.03 ± 5.24 kg/m^2^ (*p* = 0.325), and 29.72 ± 4.40 kg/m^2^ and 27.42 ± 4.47 kg/m^2^ (*p* = 0.186); no statistical relevant differences were registered between the two groups (Table 3).

In both groups, we had an excellent result in terms of comorbidity resolution; six patients in group A (86% of diabetic pts) and 4 in group B (80%) had a complete resolution of T2DM after 6 months (*p* = 0.755). Hypertension in the two groups has registered a decrease from 56% to 28% of patients in group A and from 28% to 4% in group B (*p* = 0.022). After 6 months after surgery, no patients suffered from OSAS.

No patients referred to solid food intolerance although four of them, two in group A and 2 in group B, experienced emetic episodes two times per week during the first 6 months after surgery.

## 4. Discussion

Surgery represents the only effective treatment for morbid obesity, with a significant reduction of morbidity and mortality rates and costs [11].

LSG is highly successful in the short-term follow-up while about 30–40% of patients require a second-step operation for failure [12, 13]. This phenomenon is probably linked to a slow but progressive dilation of the new stomach, and it occurs more frequently in extremely obese patients with preoperative BMI > 50.

Following the encouraging results reported by various authors [7–9, 14, 15] with the banded RYGB in the long-term weight loss maintenance, some investigators began to perform banded sleeve gastrectomy with the same principles in order to reduce gastric dilation and therefore to decrease long-term weight regain.

In 2009 Alexander et al. published a series of 27 patients submitted to LBSG fashioned with a piece of AlloDerm® and Prolene suture [5]. They compared the results obtained during a follow-up period of 12 months with a group of patients who underwent LRYGB during the same period. No significative differences were noticed between the two groups in terms of BMI reduction and comorbidities improvements/resolution.

Similarly Karcz et al. recently published the results of 25 obese patients submitted to banded sleeve gastrectomy using a synthetic MiniMizer® Ring. They made a retrospective matched-pair analysis selecting a similar number of patients previously treated with LSG at the same institution [4]. They noticed that the results between the two groups in terms of %EWL (excess weight loss) did not differ after 12 months of follow-up, while the presence of the ring increased the occurrence of vomiting.

We believe that the use of biocompatible tissues, as bovine pericardial patch, around the gastric tube is advantageous for the integration and compatibility with the body; on the other hand, this process might compromise the desired restrictive effect on the remaining stomach over time; furthermore, the integration of heterologous tissues with the gastric wall might interfere with further surgical maneuvers during reoperation if needed. For this reason, we believe that the use of a synthetic, biocompatible, light, and manageable device with an easy sealing system might result to be advantageous.

We described the first randomized prospective study comparing LSG and LBSG performed with the GaBP Ring Autolock, a silicone coated implantable ring of 6.5 to 7.5 cm diameter with a self-closing device. We reported the results with a 12-month follow-up while the long-term outcomes will be described in the future.

It is reassuring to notice that our postoperative complications following LBSG are similar to those observed after LSG; furthermore, the overall complication rate of this study is comparable with the recent literature about LSG. This supports the fact that the procedure is safe and feasible and it does not expose the patient to major risks compared to a traditional LSG.

An increasing percentage of remission of systemic hypertension registered in LBSG patients leads us to expect that, over time, this dichotomy will tend to increase rather than decrease.

The weight loss did not show any statistically significant reduction between the two groups, even though we suppose that this factor is influenced by the relatively short follow-up period.

Burton and Brown state that the weight loss in restrictive interventions is mainly due to the early satiety phenomenon rather than the degree of restriction [16], thus leading to the hypothesis that the presence of a ring in the proximal portion of the stomach can further reduce the progression of alimentary bolus and shorten the satiety reaching time in LBSG patients. Actually we did not observe any deceleration of the gastric transit time during the early swallow studies. In addition, also late swallow studies did not show any bolus slowdown suggesting the hypothesis that the ring does not represent a functional stenosis but rather a useful tool to prevent gastric dilation. This data is confirmed by the absence of food intolerance in our patients. This is important also because we can speculate that the ring should not represent a risk factor for gastric leakage in a higher pressure zone, above the ring. In other words, it seems that the ring prevents gastric dilation without producing an additional restriction.

We hypothesize that the effects related to the positioning of the ring will be more evident in the period of time generally related to the neostomach dilation, which goes from 2 to 5 years from the primary intervention.

On the basis of our results we can state that the positioning of the GaBP Ring Autolock System after LSG is a safe procedure and it does not influence the incidence of postoperative complications such as bleeding and gastric fistula.

A longer follow-up period will be necessary to provide definitive conclusion regarding long-term benefits.

## 5. Conclusions

Our findings confirm that the LBSG using the GaBP Ring Autolock is a safe and feasible procedure.

No significant differences in the incidence of postoperative complications have been found among the two groups, though slightly higher for the control group.

The time required for the device positioning did not influence significantly the surgical time. Concerning the obesity-related morbidities, both groups showed a high percentage of remission. On the other hand, the results of bodyweight loss did not document any statistically significant differences among the two groups, even though LBSG group showed a mean BMI slightly lower than that of the control group in the 6 and 12 months follow-ups. This trend, still negligible and not significant, will have to be evaluated over time, and, in case it becomes significant, it could affect the current surgical technique used to perform sleeve gastrectomy.

## Figures and Tables

**Table 1:** Demographic of patients enrolled in the study.

	Total	Group A	Group B
Number of patients	50	25	25
Sex	32 F/18 M	16 F/9 M	16 F/9 M
Mean BMI	45.99 ± 6.25 kg/m^2^	47.03 ± 6.58 kg/m^2^	44.95 ± 5.85 kg/m^2^
T2DM	12	7	5
Hypertension	21	14	7
OSAS	8	6	2

**Table 2:** Postoperatory complications.

	Group A	Group B
Total	8% (number: 2)	4% (number: 1)
Bleeding	4% (number: 1)	4% (number: 1)
Gastric stenosis	4% (number: 1)	—

**Table 3:** Mean BMI in the groups.

Follow-up	Group A	Group B	*p* value
Pre-op	47.03 ± 6.58 kg/m^2^	44.95 ± 5.85 kg/m^2^	0.244
3 months	37.86 ± 5.72 kg/m^2^	37.58 ± 6.21 kg/m^2^	0.869
6 months	33.64 ± 6.08 kg/m^2^	32.03 ± 5.24 kg/m^2^	0.325
12 months	29.72 ± 4.40 kg/m^2^	27.42 ± 4.47 kg/m^2^	0.186
